# Supplementary material for: Tuberculosis-related deaths at a tertiary hospital in Zambia: Insights into the prevalence and associated factors
Source: PLOS Glob Public Health. 2024 Oct 14;4(10):e0003686. doi: 10.1371/journal.pgph.0003686 (PMC11472957; doi:10.1371/journal.pgph.0003686)
Supplement: S1 Table — (DOCX) [file pgph.0003686.s002.docx]

| **S1 Table : Basic demographic and Clinical Characteristics among adult patients with Drug-Susceptible TB Mortality with pulmonary TB and disseminated TB** | | | | | | |
| --- | --- | --- | --- | --- | --- | --- |
|  | **Pulmonary TB** | | | **Extra pulmonary TB** | | |
| **Variable** | **Died** | **Alive** | **P value** | **Died** | **Alive** | **P value** |
|  | (n=42, 8.3 %) | (n=465, 91.7 %) |  | (n=51, 10.1%) | (n=456, 89.9%) |  |
| **Age, years** | 43.5 ( 34, 52) | 41 ( 23, 50 ) | 0.365 | 40 (32, 49) | 41 (32, 51) | 0.936 |
| **Age category, years** |  |  | 0.516 |  |  | 0.509 |
| 18-24 | 3 (6.0) | 47 (94.0) |  | 2 (4.0) | 48 (96.0) |  |
| 25-34 | 8 (7.1) | 104 (92.9) |  | 12 (10.7) | 100 (89.3) |  |
| 35-44 | 13 (8.8) | 134 (91.2) |  | 17 (11.6) | 130( 88.4) |  |
| 45-54 | 11 (10.9) | 90 (89.1) |  | 11 (10.9) | 90 (89.4) |  |
| 55-64 | 1 (2.3) | 43 (97.7) |  | 6 (13.6) | 38 (86.4) |  |
| 65 and above | 6 ( 2.3) | 47 (88.7) |  | 3 (5.7) | 50 (94.3) |  |
| **Sex** |  |  | 0.31 |  |  | 0.821 |
| Male | 26 (9.4) | 250 ( 90.6) |  | 27 (9.8) | 249 (90.2) |  |
| Female | 16 (6.9) | 215 (93.1) |  | 24 (10.4) | 207 (89.6) |  |
| **Residence** |  |  | 0.678 |  |  | 0.45 |
| Urban | 23 ( 7.9) | 270 (92.2) |  | 32 (10.9) | 261 (89.1) |  |
| Rural | 19 (8.9) | 195 (91.1) |  | 19 (8.9) | 195 (91.1) |  |
| **Occupation** |  |  | 0.326 |  |  | 1 |
| Health care worker | 1 (16.7) | 5 (83.3) |  | 0 (0.0) | 6 (100.0) |  |
| Miner | 1 (12.5) | 7 (87.5) |  | 1 (0.0) | 8 (100.0) |  |
| Others | 40 (8.1) | 453 (81.9) |  | 51 (10.3) | 442 (89.7) |  |
| **PLWH,** n=506 |  |  | 0.166 |  |  | 0.086 |
| Yes | 30 (9.6) | 281 (90.4) |  | 14 (7.2) | 181 (92.8) |  |
| No | 12 (6.2) | 183 (93.9) |  | 37 (11.9) | 274 (88.1) |  |
| **Presumptive CPT,** n=311 |  |  | 0.661 |  |  | 0.647 |
| Yes | 28 (9.7) | 262 (90.3) |  | 34 (18.6) | 149 (81.4) |  |
| No | 1 (6.3) | 15 (93.7) |  | 2 (22.2) | 7 (77.8) |  |
| missing/unknown | 1 (20.0) | 4 (80.0) |  | 2 (33.3) | 4 (66.7) |  |
| **Patient type** |  |  | 0.412 |  |  | 0.863 |
| New | 30 (8.1) | 340( 91.9) |  | 37 (10.0) | 333 (90.0) |  |
| Relapse | 11 (11.1) | 88 (88.9) |  | 11 (11.1) | 88 (88.9) |  |
| Treatment after loss to follow up | 1 (5.0) | 19 (1.1) |  | 1 (5.0) | 19 (95.0) |  |
| other category | 0 (0.0) | 17 (100.0) |  | 2 (11.8) | 15 (88.2) |  |
| **Weight at Start of treatment, kg,** n=491 | 46 (43, 61) | 54 (46, 61 ) | 0.0523 | 48 (44.8, 56) | 54 (45 , 62) | **0.026** |
| **Weight at end of treatment,kg,** n=361 | 56.5 (38 , 75 ) | 58 (50, 67) | 0.817 | 45.5 ( 40, 56.5) | 58 (50, 67) | 0.082 |
| **DOT plan,** n=482 |  |  | **0.004** |  |  | **<0.001** |
| Observed daily at clinic | 31 (11.5) | 239 (88.5) |  | 37 (13.7) | 233 (86.3) |  |
| Observed daily by family | 9 (4.3) | 203 (95.7) |  | 10 (4.7) | 202 (95.3) |  |
| **Treatment outcome** |  |  |  |  |  |  |
| Cured |  |  |  |  |  |  |
| Died |  |  |  |  |  |  |
| lost to follow up |  |  |  |  |  |  |
| Treatment failure |  |  |  |  |  |  |
| **HB, *g/dL,*** *n=180* | 9.6 (8.6, 12.3 ) | 10.9 (8.5, 13.0) | 0.346 | 9.1 (5.7, 9.8) | 11.3 (9.0, 13.0) | **< 0.001** |
| **Wbc, *10^9/L,*** *n=177* | 8.6 (5.6, 11.2) | 5.8 ( 4.1, 8.5) | 0.065 | 6.5 (4.1, 9.5) | 5.8 (4.2, 8.5) | 0.592 |
| **Creatinine, *µmol/L,*** *n=99* | 123.8 (103.2 ,184.7) | 91.8(78.0, 113.8) | 0.061 | 87.3 (68.9, 149.8) | 92.1 (81.5, 117.7) | 0.584 |
| **Urea, *mg/dL,*** *n= 73* | 5.3 (3.7, 11.9) | 3.9 (3.0, 6.3 ) | 0.139 | 10.5 (6.8, 18.9) | 3.9 (2.9, 6.3) | **0.006** |
| **Platelet, *10^9/L,*** *n=174* | 237 (126, 306) | 266 ( 193, 385) | 0.201 | 264 (197, 329.5) | 268 (189, 385) | 0.691 |
| **ALT, *IU/L,*** *n=63* | 27.5 (17.8, 52.8) | 21.4 (15, 33.2) | 0.535 | 22.6 (14.2, 27.2) | 21.9 (15.2, 33.2) | 0.367 |
| **AST, *IU/L,*** *n=80* | 52.7 ( 40.1, 704 ) | 32.4 ( 27.5, 47.1 ) | 0.059 | 33.7 (31.7, 54.1) | 35.4 (26.7, 47.1) | 0.638 |
| **CD4 count, *cells/µL,*** *n=71* | 111.5 (49, 270.5) | 378 (180, 599) | 0.065 | 185.0 (37, 424) | 394 (205.0, 599.0) | 0.07 |
| **HIV VL, copies ,** n=20 |  |  |  |  |  |  |
| **Note**: 203 TB cases were diagnosed clinically and the rest were bacteriologically confirmed , cotramoxazale was given to HIV patient only | | | | | | |
| **Abbreviation :**HB (hemoglobin),CPT (Cotrimoxazole preventive therapy) , WBC (white blood cells), ALT (Alanine transaminase) , AST(Aspartate transaminase), DOT( Direct observation therapy , others( other profession category), TB (tuberculosis), PLWH (People living with HIV) | | | | | | |
