# Supplementary material for: Tuberculosis-related deaths at a tertiary hospital in Zambia: Insights into the prevalence and associated factors
Source: PLOS Glob Public Health. 2024 Oct 14;4(10):e0003686. doi: 10.1371/journal.pgph.0003686 (PMC11472957; doi:10.1371/journal.pgph.0003686)
Supplement: S2 Table — (DOCX) [file pgph.0003686.s003.docx]

| S2 Table: Basic demographic and Clinical Characteristics among pediatrics patients with Drug-Susceptible TB Mortality with pulmonary TB and disseminated TB | | | | | | | |
| --- | --- | --- | --- | --- | --- | --- | --- |
|  |  | **Pulmonary TB** | |  | **Disseminated TB** | | |
| Variable | **Median, (IQR) OR Frequency (%)** | **Died** | **Alive** | **P value** | **Died** | **Alive** | **P value** |
|  |  | 7 (3.7%) | 180 (96.3) |  | 6 (3.2) | 181 (96.7) |  |
| Age, years | 2 (1, 8) | 16 ( 10, 17) | 2 ( 1, 7) | **0.002** | 0.7 ( 1, 7) | 2 (1, 8) | 0.346 |
| Age category, years |  |  |  | **0.001** |  |  | 0.965 |
| 0 – 4 | 115 (61.5) | 1 (0.87) | 114 (99.1) |  | 4 (3.5) | 111 (96.5) |  |
| 5- 11 | 37 (19.8) | 1 (5) | 36 (97.3 ) |  | 1 (2.7) | 36 (97.3) |  |
| 12-18 | 35 (18.7) | 5 ( 14.3 ) | 30 (85.7) |  | 1 (2.9) | 34 (97.1) |  |
| Sex |  |  |  | 0.934 |  |  | 0.778 |
| Male | 104 (55.6) | 4 (3.9) | 100 (96.2) |  | 3 (2.9) | 101 (97.1) |  |
| Female | 83 (44.4) | 3 (3.6) | 80 (96.3) |  | 3 (2.6) | 80 (96.4) |  |
| Residence |  |  |  | 0.242 |  |  | 0.989 |
| Urban | 94 (50.3) | 2 (2.1 ) | 92 (97.9) |  | 3 (3.2) | 91 (96.8) |  |
| Rural | 93 (49.7) | 5 (5.4) | 88 (94.6) |  | 3 (3.2) | 90 (96.8) |  |
| PLWH, n=175 |  |  |  | **0.002** |  |  | 0.534 |
| Yes | 135 (77.1) | 5 (12.5) | 35 ( 87.5 ) |  | 4 (3.0) | 131 (97.0) |  |
| No | 40 (22.9) | 2 (1.5) | 133 (98.5) |  | 2 (5.0) | 38 (95.0) |  |
| Presumptive CPT, n=183 |  |  |  | **0.002** |  |  | 0.943 |
| Yes | 36 (90.0) | 2 (5.6) | 34 (94.4) |  | 2 (5.6) | 34 (94.4) |  |
| No | 1 (2.5) | 0 (0.0) | 1 (100) |  | 0 (0.0) | 1 (100.0) |  |
| missing/unknown | 3 (7.5) | 1 (100) | 0 (0.00) |  | 0 (0.0) | 1 (100.0) |  |
| Patient type |  |  |  | 0.856 |  |  | 0.684 |
| New | 169(90.4 ) | 7 (4.1) | 162 (95.9) |  | 5 (3.0) | 164 (97.0) |  |
| Relapse | 11 (5.9) | 0 (0.0) | 11 (100.0) |  | 1 (9.1) | 10 (90.9) |  |
| Treatment after loss to follow up | 3 (1.6) | 0 (0.0) | 3 (100.0) |  | 0 (0.0) | 3 (100.0) |  |
| other category | 4 (2.1) | 0 (0.0) | 4 (100.0) |  | (0. 0) | 4 (100.0) |  |
| Weight at Start of treatment, kg, n=185 | 9.2 (6.8) |  |  |  |  |  |  |
| Weight at end of treatment,kg, n=138 | 13.2 (9.4, 24.3) |  |  |  |  |  |  |
| DOT plan, n=184 |  |  |  | 0.33 |  |  | 0.441 |
| Observed daily at clinic | 127 (69.0) | 6 ( 4.7) | 121 (95.3 ) |  | 5 (3.9) | 122 (96.1 ) |  |
| Observed daily by family | 57 (31.0)) | 1 (1.8) | 56 (98.3 ) |  | 1 (1.8) | 56 (98.3 ) |  |
| Treatment outcome |  |  |  |  |  |  |  |
| Cured | 173 (93.0) |  |  |  |  |  |  |
| Died | 13 (7.0) |  |  |  |  |  |  |
| lost to follow up | 2 (0.5) |  |  |  |  |  |  |
| Treatment failure | 1 (1.0) |  |  |  |  |  |  |
| Abbreviation :HB (hemoglobin),CPT (Cotrimoxazole preventive therapy) , WBC (white blood cells), ALT (Alanine transaminase) , AST(Aspartate transaminase), DOT( Direct observation therapy , others( other profession category), TB (tuberculosis), PLWH (People living with HIV)  Note: 203 TB cases were diagnosed clinically and the rest were bacteriologically confirmed | | | | | | | |
