# Supplementary material for: Tuberculosis-related deaths at a tertiary hospital in Zambia: Insights into the prevalence and associated factors
Source: PLOS Glob Public Health. 2024 Oct 14;4(10):e0003686. doi: 10.1371/journal.pgph.0003686 (PMC11472957; doi:10.1371/journal.pgph.0003686)
Supplement: S3 Table — (DOCX) [file pgph.0003686.s004.docx]

| S3 Table: Univariable and Multivariable logistic regression associated with mortality among adult patients with pulmonary TB and Disseminated TB | | | | | | | | |
| --- | --- | --- | --- | --- | --- | --- | --- | --- |
|  | **Pulmonary TB** | | | | **Disseminated TB** | | | |
| Variable | **OR (95%)** | **P-value** | **AOR (95%, Cl)** | **P-value** | **OR (95%)** | **P-value** | **AOR (95%, Cl)** | **P-value** |
| Age, years | 0.99 (0.98, 1.02) | 0.465 | 1.01 (0.98, 1.03) | 0.377 | 0.99 (0.98, 1.02) | 0.798 | 0.99 (0.97, 1.02) | 0.696 |
| Sex |  |  |  |  |  |  |  |  |
| Female | Ref |  | ref | 0.197 | ref |  | Ref |  |
| Male | 1.32 (0.73, 2.67) | 0.312 | 1.57 ( 0.79, 3.13) |  | 0.94 (0.52, 1.67) | 0.821 | 1.21 (0.64, 2.28) | 0.261 |
| PLWH |  |  |  | 0.138 |  |  |  |  |
| No | Ref |  | ref |  | ref |  | Ref |  |
| Yes | 1.62 (0.81, 3.26) | 0.169 | 1.74 ( 0.83, 3.65) |  | 1.72 (0.91, 3.32) | 0.089 | 1.44 (0.73, 2.80) | 0.283 |
| DOT plan |  |  |  |  |  |  |  |  |
| Observed daily at clinic | Ref |  | ref |  | ref |  | Ref |  |
| Observed daily by family | 0.34 (0.16, 0.73) | **0.006** | 0.33 ( 0.15, 0.72) | **0.005** | 0.31 (0.15, 0.64) | 0.002 | 0.32 (0.15, 0.66) | **0.002** |
| Abbreviation: DOT (Direct observation therapy, PLWH (People living with HIV) | | | | | | | | |
