# Supplementary material for: Tuberculosis-related deaths at a tertiary hospital in Zambia: Insights into the prevalence and associated factors
Source: PLOS Glob Public Health. 2024 Oct 14;4(10):e0003686. doi: 10.1371/journal.pgph.0003686 (PMC11472957; doi:10.1371/journal.pgph.0003686)
Supplement: S4 Table — (DOCX) [file pgph.0003686.s005.docx]

| S4 Table: Univariable and Multivariable logistic regression associated with mortality among pediatric patients with pulmonary TB and Disseminated TB | | | | | | | | |
| --- | --- | --- | --- | --- | --- | --- | --- | --- |
|  | **Pulmonary TB** | | | **Disseminated TB** | | | | |
| Variable | **OR (95%, Cl** | **P-value** | **AOR (95%, Cl)** | **P-value** | **OR (95%)** | **P-value** | **AOR (95%, Cl)** | **P-value** |
| Age, years |  |  |  |  |  |  |  |  |
| 0 – 4 | ref |  | ref |  |  |  | ref |  |
| 5- 11 | 3.17 ( 0.19, 51.9) | 0.419 | 2.09 (0.11, 40.8) | 0.626 | ref |  | 0.85 (0.08 , 8.8) | 0.889 |
| 12-18 | 19 ( 2.13, 168.8 ) | 0.008 | 31.9(2.61 , 392.9) | **0.007** | 0.77(0.08, 7.1) | 0.819 | 0.80 (0.08, 7.9) | 0.852 |
| Sex |  |  |  |  | 0.81 (0.088, 7.5 | 0.858 |  |  |
| Female | Ref |  | ref |  | ref |  | ref |  |
| Male | 0.93 (0.20 , 4.13) | 0.312 | 2.49(0.33, 18.8) | 0.376 | 0.79(0.2, 5.0) | 0.779 | 0.89( 0.2 , 4.7) | 0.893 |
| PLWH |  |  |  |  |  |  |  |  |
| No | Ref |  | ref |  | ref |  | ref |  |
| yes | 9.5 (1.76, 51.1) | 0.009 | 22.2(2.21, 222.8) | **0.008** | 1.7 (0.3, 9.8) | 0.539 | 1.93( 0.3, 11.8) | 0.475 |
| DOT plan |  |  |  |  |  |  |  |  |
| Observed daily at clinic | Ref |  | ref |  | ref |  | ref |  |
| Observed daily by family | 0.36 (0.042, 3.06) | 0.35 | 0.07(0.01, 0.91) | **0.043** | 0.43 (0.04, 3.81) | 0.453 | 0.4 (0.04, 3.8) | 0.431 |
| Abbreviation: DOT (Direct observation therapy, PLWH (People living with HIV) | | | | | | | | |
