# Supplementary material for: Tuberculosis-related deaths at a tertiary hospital in Zambia: Insights into the prevalence and associated factors
Source: PLOS Glob Public Health. 2024 Oct 14;4(10):e0003686. doi: 10.1371/journal.pgph.0003686 (PMC11472957; doi:10.1371/journal.pgph.0003686)
Supplement: Attachment — Submitted filename: PGPH-24-01455 REVIEW (2).pdf [file pgph.0003686.s007.pdf]

Thank you for asking me to review this manuscript.

It is well written with a touch of seniority in academic writing and informative.

TB related mortality and morbidity are one of the biggest public health challenges especially in low and middle income countries. Data to inform clinicians about regional predictors of TB related mortality is of great value.

Abstract: The abstract is well written

Introduction: the background of the problem is well stated, especially he high prevalence of TB in Zambia.

However, this section lacks novelty. What in particular this retrospective study would show.

There is data with proven risk factors for TB infection, disease, severe disease and poor treatment outcomes (death in particular). What this study will show is not clearly brought out.

In my view, clinicians in Zambia could build on prior data as shown above to improve care.

It would have been better for the study to focus on one form of TB. For example, mortality from pulmonary TB is generally about 5-10% globally. Mortality due to TBM meningitis, the most severe form of TB is up to 60% in sub-Saharan Africa, especially among the HIV+, and that of drug-resistant TB is about 40%., yet they all have different factors that lead to severe outcomes.

Methods.

1. The duration of the records is missing. We know that TB treatment guidelines, especially for drug-resistant TB have changed overtime, and this has had an effect on the outcomes.
2. TB cases; did you exclude those with a radiological diagnosis? And why?
3. Line 64, this should be in your results. Here just tell us about the inclusion and exclusion criteria.
4. I note that you excluded participants with missing key variables (age) and treatment outcomes. Did all the other participants have complete data? What was the % of missing data, to have a participant/medical record excluded?
5. Why consider Cotrimoxazole as pre-emptive therapy and not mention ART status, CD4 count, Smoking status, alcohol consumption, TB preventive therapy, etc. What was special with Cotrim?
6. Can you describe the nature of the medial records, is it the ECBSS, Open MRS or paper records, given the short duration of data collection?
7. The treatment outcomes were updated in 2021. See Nguyen Nhat et al, 2021, ERS, editorial.

Results:

1.I am still concerned about missing data. It is hard to believe that there was no missing data in a retrospective chart review, and this wasn't considered in analysis.

2. Please re-write line 132-33, remove the wording respectively.

3. No mention of ART status and CD4/viral loads. Is the TB program not integrated with HIV care services in Zambia?

4. Since you used the chi-square test, what was the p-value, line 149?

5. line 149-150, you need to include data mentioned in 3 above to make this a more meaningful paper.

6. You cannot group minors and adults when looking for predictors of mortality.

7. In the text PLWH are 351, in the table, PLWH, has an n=681, this needs to be re-organised

8. The table subheadings has TB related deaths, but treatment outcome as one of the variables. Can this be moved?

9. The laboratory tests, did all patients have these tests? Any with missing data?

10. Table 2: Why are males the reference category yet they are biologically known and well documented to have poor TB outcomes?

11. You need CD4 counts, ART status to justify PLWH deaths (Table 2_) You had this as a variable in your data collection tool (see data set, AI, AJ), but you didn't collect it, why?

12. The dataset shows more patients seen at the Pead clinic than chest clinic. Can you describe in your methodology why your study site was primarily at the chest clinic but you have children? also, which population is classified as paediatric to justify your numbers?

13. I would recommend re-analysing your data. For example, you had 310, 45% with extrapulmonary TB, not 282 as shown in your table. Also, the sex codes are messed up at some point. That's why you have 380 males, and when coded to 1, you have 382.

Discussion.

Generally, well written.

1. This study had a slightly higher mortality, not because of selection bias as stated, but due to inclusion of all types of Tb without separating them and all age groups. Also, the study cited line 173 was conducted in 2016 and published in 2021, not 2024 as stated in your text.
2. Line182, if the immunity was of great concern, then children would also die more. Can you find another way to explain this relationship? Is it that these are the same people that presented late, were HIV co-infected, etc?
3. Line 188, you need to defend this with the immune status.
4. Why was and isn't verbal autopsy done? This is part of the ecbss, and open mrs.
5. The section of recommendations is equally missing.

Generally, the manuscript LACKS novelty and has major gaps that need to be filled. I will leave the recommendation to accept or reject the manuscript at the discretion of the editor in chief.

Reviewed by

Edwin Nuwagira
