# Supplementary material for: Tuberculosis-related deaths at a tertiary hospital in Zambia: Insights into the prevalence and associated factors
Source: PLOS Glob Public Health. 2024 Oct 14;4(10):e0003686. doi: 10.1371/journal.pgph.0003686 (PMC11472957; doi:10.1371/journal.pgph.0003686)
Supplement: Attachment — Submitted filename: Response to Reviewers.docx [file pgph.0003686.s008.docx]

**Responses to Reviewers' comments:**

Reviewer #1: Thanks for allowing me to review this interesting manuscript. It is a well written manuscript

I have the following minor comments

1.Line 70. specify that cotrimoxazole was used for only those patients who are HIV positive

**Response:** Thank you very much for the correction. We have now specified

2. Cross check line 110 to 111 for correct sentence construction ..... then excel...the cleaned....

**Response:** Thank you very much for the observation. We have changed the text construction.

3. Include a figure showing data abstraction flow

**Response:** Thank you very much. Data abstraction flow has been added.

4. Line 140; a sentence should not start with a figure
**Response: thank you. We have revised this**

5. The reference study cited in line 173 was done in 2021 not 2024

**Response:** Thank you very much for the correction. The text has been cited appropriately.

**Reviewer # 2**

Thank you for asking me to review this manuscript. It is well written with a touch of seniority in academic writing and informative. TB related mortality and morbidity are one of the biggest public health challenges especially in low and middle income countries. Data to inform clinicians about regional predictors of TB related mortality is of great value.

**Abstract:** The abstract is well written

**Response:** thank you.

**Introduction**: the background of the problem is well stated, especially the high prevalence of TB in Zambia. However, this section lacks novelty. What in particular this retrospective study would show.

There is data with proven risk factors for TB infection, disease, severe disease and poor treatment outcomes (death in particular). What this study will show is not clearly brought out. In my view, clinicians in Zambia could build on prior data as shown above to improve care. It would have been better for the study to focus on one form of TB. For example, mortality from pulmonary TB is generally about 5-10% globally. Mortality due to TBM meningitis, the most severe form of TB is up to 60% in sub-Saharan Africa, especially among the HIV+, and that of drug-resistant TB is about 40%., yet they all have different factors that lead to severe outcomes.

**Response:** thank very much for the suggestion. To improve the manuscript were have split the data into adult and pediatrics. Then we have further analyzed the data among the two groups based on the form of TB (pulmonary and extra pulmonary). This is now included in the supplementally file

**Methods.**

1. The duration of the records is missing. We know that TB treatment guidelines, especially for drug-resistant TB have changed overtime, and this has had an effect on the outcomes.

**Response:** thanks you for the correction. We have now specified the duration of records in the study design and setting section under the methodology

2. TB cases; did you exclude those with a radiological diagnosis? And why?

**Response**: thank you. We didn’t exclude patient with radiological diagnosis all patients during the records review were included regardless of the means of diagnosis used.

3. Line 64, this should be in your results. Here just tell us about the inclusion and exclusion criteria.

**Response:** thank you for the correction. The sentences has been revised

4. I note that you excluded participants with missing key variables (age) and treatment outcomes. Did all the other participants have complete data? What was the % of missing data, to have a participant/medical record excluded?

**Response**: thank you very much for this observation. Not all participants had complete data especially laboratory investigations. We mainly excluded those who we could not determine the treatment outcome and age

5. Why consider Cotrimoxazole as pre-emptive therapy and not mention ART status, CD4 count, Smoking status, alcohol consumption, TB preventive therapy, etc. What was special with Cotrim?

**Response:**  Cotrimoxazole is implemented as an integral component of the care cascade as preventative therapy. While we have included CD4 and viral loads etc., the data records did not have specific ART regimens for those who were HIV mainly because the TB clinic has not integrated the ART program and this is because not all TB patients will have HIV. We have anow included this limitation in the discussion.

6. Can you describe the nature of the medial records, is it the ECBSS, Open MRS or paper records, given the short duration of data collection?

**Response**: thank you. We used paper-based treatment register for TB patient which is used at them facility were have include this information in the data collection section.

7. The treatment outcomes were updated in 2021. See Nguyen Nhat et al, 2021, ERS, editorial.

**Response:** Thank you for the correction. The treatment outcome have been changed according to the updated guidelines

**Results**:

1.I am still concerned about missing data. It is hard to believe that there was no missing data in a retrospective chart review, and this wasn’t considered in analysis.

**Response:** the tables of results are now clearly showing missing data for each variable. The “n” has been included for each variable for this purpose.

2. Please re-write line 132-33, remove the wording respectively.

**Response:** thank you for the correction the word respectively has been removed.

3. No mention of ART status and CD4/viral loads. Is the TB program not integrated with HIV care services in Zambia?

**Response: thank you.** The CD4/Viral loads have now been added to the table. The majority of patients receiving TB treatment at our facility receive their HIV management from their local facility, which maintains their actual ART status and CD4/viral loads. Since there is currently no central system to access these records, tracing these records from multiple ART centers has proven challenging. With the exception of a few patients, the physicians at our facility had orders for these CD4/viral tests.

4. Since you used the chi-square test, what was the p-value, line 149?

**Response:** thank you for correction. WE have reanalyzed the data and indicated

5. line 149-150, you need to include data mentioned in 3 above to make this a more meaningful paper.

**Reaponse**: thank you. We have done so as explained in 3 above

6. You cannot group minors and adults when looking for predictors of mortality.

**Response:** thank you. We have split the minors and adults and analyzed the data separately

7. In the text PLWH are 351, in the table, PLWH, has an n=681, this needs to be re-organised

**Response:** thank you. The error has been rectified

8. The table subheadings has TB related deaths, but treatment outcome as one of the variables. Can this be moved?

**Response: Thank you. We have done as advised.**

9. The laboratory tests, did all patients have these tests? Any with missing data?

**Response**: thank you. They were missing laboratory tests. We have added a column on the table to show the variables analyzed.

10. Table 2: Why are males the reference category yet they are biologically known and well documented to have poor TB outcomes?

**Response:** thanks for the correction. The reference group has been changed to females.

11. You need CD4 counts, ART status to justify PLWH deaths (Table 2_) You had this as a variable in your data collection tool (see data set, AI, AJ), but you didn’t collect it, why?

**Response:** thank you. see 3 above.

12. The dataset shows more patients seen at the Pead clinic than chest clinic. Can you describe in your methodology why your study site was primarily at the chest clinic but you have children? also, which population is classified as paediatric to justify your numbers?

**Response:** thank you. We have now put a justification and clearly outlined this in the methodology section. The chest clinic is the primary place, which manages all patients of all ages diagnosed with TB and leprosy

13. I would recommend re-analysing your data. For example, you had 310, 45% with extra pulmonary TB, not 282 as shown in your table. Also, the sex codes are messed up at some point. That’s why you have 380 males, and when coded to 1, you have 382.

**Response**: thank you for the suggestion. We have reanalyzed the data

**Discussion**. Generally, well written.

1. This study had a slightly higher mortality, not because of selection bias as stated, but due to inclusion of all types of Tb without separating them and all age groups. Also, the study cited line 173 was conducted in 2016 and published in 2021, not 2024 as stated in your text.

**Response:** thank you, we have desegregated the data into age group into adult and minors and reanalyzed the data. Then in the we have also further reanalyzed the data based on the form of TB and age and put them in the supplementary file.

2. Line182, if the immunity was of great concern, then children would also die more. Can you find another way to explain this relationship? Is it that these are the same people that presented late, were HIV co-infected, etc?

**Response:** thank you for the suggestion. We have rewritten the sentence for clarity.

3. Line 188, you need to defend this with the immune status.

**Response:** thank you for the suggestion. We have rewritten the sentence and defended with immune status.

4. Why was and isn’t verbal autopsy done? This is part of the ecbss, and open mrs.

Response: the nature of the retrospective data using paper-based records could not allow collecting additional data as suggested.

5. The section of recommendations is equally missing. Generally, the manuscript LACKS novelty and has major gaps that need to be filled. I will leave the recommendation to accept or reject the manuscript at the discretion of the editor in chief.

**Response:** thank you. We have put the recommendation as part of the conclusion.
